# Flavonoid intake is inversely associated with obesity and C-reactive protein, a marker for inflammation, in US adults

**DOI:** 10.1038/nutd.2017.22

**Published:** 2017-05-15

**Authors:** J A Vernarelli, J D Lambert

**Affiliations:** 1 Department of Biology, Fairfield University, Fairfield, CT, USA; 2 Marion Egan Peckham School of Nursing and Health Studies, Fairfield University, Fairfield, CT, USA; 3 Department of Food Science, The Pennsylvania State University, University Park, PA, USA

## Abstract

Recent studies have demonstrated the importance of flavonoid intake and disease risk, however the association between flavonoid intake and obesity has not been evaluated in a nationally representative sample of US adults. The objective of the study was to evaluate the association between flavonoid consumption and established risk factors for obesity and obesity-related inflammation. Data from a nationally representative sample of 9551 adults who participated in the 2005–2008 National Health and Nutrition Examination Survey (NHANES) were analyzed. Flavonoid consumption was inversely associated with obesity in both men and women in multivariate models. Adults in the highest quartile of flavonoid intake had significantly lower body mass index and waist circumference than those in the lowest quartile of flavonoid intake (*P*<0.03 and *P*<0.04, respectively), and flavonoid intake was inversely related to C-reactive protein levels in women (p-trend, 0.01). These findings support a growing body of laboratory evidence that flavonoid consumption may be beneficial for disease prevention.

## Introduction

Over the past few decades, the rates of obesity have risen markedly.^1^ Overweight and obesity have been identified as risk factors for several diseases, including cardiovascular disease, diabetes and cancer. Survey data from large, nationally representative samples of US individuals can provide insight into trends that have potentially contributed to this increase in obesity. Data from the National Health and Nutrition Examination Surveys (NHANES) have shown that although there have been no substantial changes in eating frequency, snacking behaviors or overall energy intake among US adults, there have been significant changes in macronutrient distribution and beverage consumption, suggesting that specific dietary patterns, such as increased intake of carbohydrates and sugar-sweetened beverages, may influence weight status.^2, 3, 4^

Flavonoid consumption has been linked to a decrease in risk for: stroke;^5^ cardiovascular disease;^6, 7^ asthma;^8^ and some cancers.^9, 10, 11, 12, 13, 14^ The majority of studies on the relationship between flavonoid intake and obesity have focused on intake of specific flavonoids subtypes and body mass index or weight gain^15, 16^ or lipid metabolism;^17^ with very few assessing the relationship between overall flavonoid intake and obesity. The objective of this study was to determine the relationship between flavonoid consumption and obesity in a representative sample of 9551 US adults. Using NHANES data, the association of flavonoid consumption and multiple markers for obesity including: body mass index (BMI), waist circumference and C-reactive protein were explored.

## Materials and methods

### Data source

NHANES is a large, cross-sectional survey conducted by National Center for Health Statistics. NHANES is designed to monitor the health and nutritional status of non-institutionalized civilians in the US; nationally representative survey and physical data are collected on a continual basis and released in two-year increments. Complete details regarding the NHANES sampling methodology, data collection, and interview process are available on the NHANES website (http://www.cdc.gov/nchs/nhanes.htm). Written consent is obtained from all NHANES participants. Data from the 2005–2008 survey cycles were combined for this study. The present study was approved by the Institutional Review Board at the Pennsylvania State University.

### Anthropometric and biomarker data

In both cycles of NHANES, height and weight were measured by trained examiners using standardized protocols and calibrated equipment during the physical examination component of the study. Adults were classified as lean (BMI ⩽24.9 kg m^−2^), overweight (BMI of 25.0–29.9) or obese (BMI ⩾30) using CDC cut points (https://www.cdc.gov/obesity/adult/defining.html). For this analysis, underweight (BMI <18.5) participants were included in the lean category. Blood samples were collected on a smaller subset of the population. Non-fasting samples were obtained for C-reactive protein (CRP), a marker for inflammation.

### Assessment of flavonoid intake

Flavonoid content of the diet was assessed using the USDA Flavonoid database version 3.0,^18^ which includes flavonols, flavones, flavanones, flavan-3-ols and anthocyanidins. The database contains a list of foods and the currently available flavonoid compound data by flavonoid class. The food data were aggregated where possible to match USDA National Nutrient Databank (NDB) standard reference (SR) codes.^18^ The SR codes were used to identify and match foods from the flavonoid database to corresponding FNDDS codes in order to identify foods that contain bioactive compounds (for example, a smoothie containing pomegranate juice). Using the FNDDS ingredient file, the gram weight of each flavonoid-containing component of a dish was determined; SR codes were used to re-link flavonoid content to corresponding 8-digit USDA food codes in the present NHANES database. The USDA has since released an expanded Flavonoid database, version 3.2, which contains data for 29 individual flavonoid compounds in six subclasses of flavonoids for every food in a subset of 2926 food items which provide the basis for the newer Food and Nutrient Database for Dietary Studies which can be used with later iterations of NHANES.^19^

### Statistical analysis

For the present analyses, we initially included all adults age 18 and older that had complete dietary and anthropometric data. Individuals who were currently following a weight-loss diet, individuals with implausible or very unusual dietary recall (for example, reporting no beverages during the 24-h recall period) and women who were pregnant or lactating were excluded, resulting in a full sample of 9551 adults. The 2005–2006 NHANES dietary data includes a food-frequency questionnaire (FFQ) in addition to 24-h recalls. In this analytic sample, only FFQ data for 4296 adults was available. Data from the FFQ was used to assess the relationship between regular consumption of flavonoid-containing foods and C-reactive protein, an inflammatory marker.

Age at the time of exam, education level, smoking status (current, former, never smoker), physical activity (measured in minutes of physical activity at a specific MET level), race and socioeconomic status were all provided in the NHANES data set. Socioeconomic status was quantified as a continuous variable using poverty–income ratio (PIR), or the ratio of family income to family-size specific poverty threshold.

All data were analyzed using SAS version 9.3 (SAS Institute, Cary, NC, USA). Specific survey procedures were used in the analysis to account for sample weights, unequal selection probability, and clustered design. Multivariate regression was used to evaluate the association of flavonoid intake with obesity (for example, body mass index, waist circumference), and markers for inflammation (C-reactive protein). Sex-specific analysis was conducted to take into account the natural differences in body composition and caloric needs between men and women. All models were adjusted for age, race, education, physical activity, smoking status, income (measured by PIR), dieting status, total alcohol intake, and caloric intake with significance determined at *P*<0.05.

## Results

**Figure 1:**
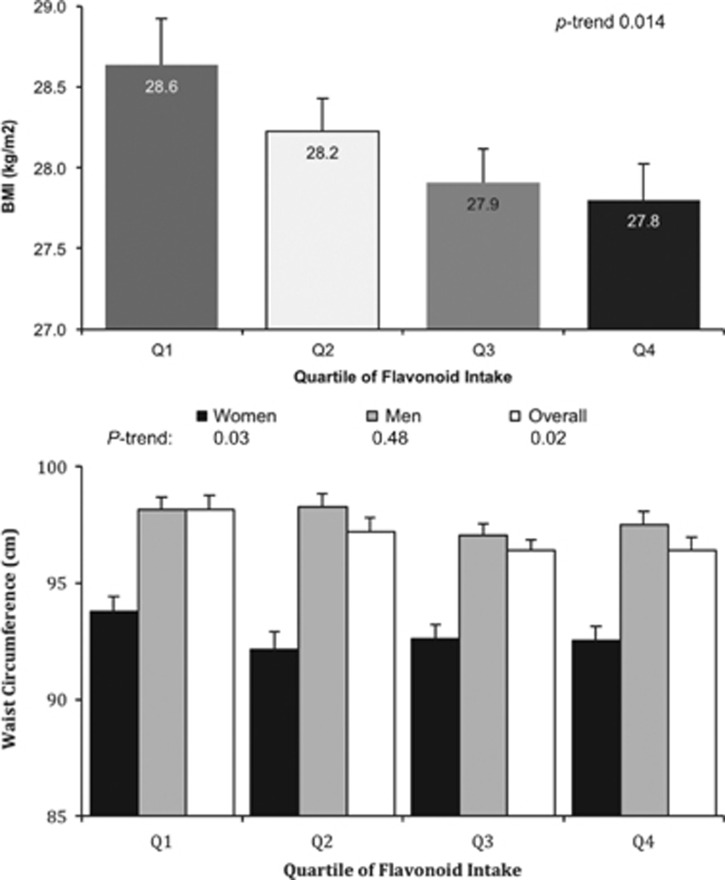
Body mass index and waist circumference by flavonoid intake quartile. Least-squared means calculated with adjustment for age, sex, race, education, physical activity, smoking status, poverty–income ratio, total alcohol intake, total fat intake and dietary energy density.

Demographic characteristics are presented in Table 1. The population sample contained equal percentages of men and women; approximately half of the sample had a history of smoking, and over 90% of the population reported consumption of a flavonoid- containing foods. An inverse association between total flavonoid intake and BMI was observed (p-trend, 0.013) after adjusting for age, sex, race, education, physical activity, smoking status, poverty:income ratio, total alcohol intake, total fat intake, and dietary energy density. (Figure 1). When evaluating flavonoid intake and waist circumference, a similar association was observed (p-trend 0.02, Figure 1). Table 2 demonstrates the relationship between regular consumption of flavonoid-containing foods and C-reactive protein levels, after controlling for the same cofactors. Across all food categories, it was noted that higher intake of flavonoid-containing foods was associated with lower CRP levels. When evaluating the association between overall flavonoid intake and CRP levels, an overall inverse association was observed (p-trend 0.01), but mean CRP levels did not differ between specific quartiles after controlling for relevant covariates.

## Discussion and conclusion

In this nationally representative sample of US adults, intake of dietary flavonoids was inversely associated with obesity and CRP. Few studies have evaluated or identified specific dietary patterns that are related to obesity and markers for Metabolic syndrome.^19^ The association between intake of a specific flavonoid-containing food (that is, tea, soy) and obesity has been previously demonstrated in a nationally representative sample of US adults,^20, 21^ has been inversely correlated to longitudinal weight gain in larger cohort studies^22^ and has also been recently correlated with lower levels of inflammatory markers in a cohort sample from the Framingham Offspring Study.^23^ However the present study takes a novel approach to assessing the relationship between total dietary flavonoid intake and markers for obesity by applying the USDA Flavonoid database to NHANES survey data. These findings support a growing body of laboratory evidence that flavonoid consumption may be beneficial for disease prevention.

## Figures and Tables

**Table 1:** Demographic characteristics of US Adults: NHANES 2005–2008

	*Sample* n^a^	*Percent*^b^
*Sex*		
Female	4587	50.0
Male	4964	50.0
*Age group*		
18–30	2270	23.5
31–50	2972	37.6
51–70	2712	27.7
>70	1597	11.2
*Race*^c^		
NH-White	4502	71.0
NH-Black	2156	11.5
Mex-Am	1802	8.3
Other	1091	9.3
*Education*		
HS or less	2931	19.8
High School Grad/GED	2427	26.5
Some College or AA degree	2545	30.0
College Graduate or above	1642	23.7
*Income*^d^		
PIR <130%	3356	24.9
130<PIR<350%	3483	34.4
PIR >350%	2712	40.7
*Smoking status*		
Never smoker	4540	50.9
Current smoker	2029	25.1
Ever smoker (>100 cigarettes)	2246	23.9
*Weight status*^e^		
Lean (BMI <25)	3259	36.2
Overweight (BMI 25–30)	3183	32.9
Obese (BMI >30)	3109	30.9
*Survey cycle*		
2005–2006	4296	48.2
2007–2008	5255	51.8
*Flavonoid consumer*		
No	434	4.9
Yes	9482	95.6

Abbreviations: BMI, body mass index; PIR, poverty–income ratio.

a Sample *n* represents raw participant counts.

b Population percentages based on NHANES survey weights and represents that population of non-institutionalized US adult residents.

c Race categories: NH-White, Non-Hispanic white; NH-Black, Non-Hispanic black, Mex-Amer, Mexican American; Other.

d Adjusted income level based on poverty:income ratio adjusted for household size and

e Weight status categorized by body mass index, measured in kg m^−^^2^.

**Table 2:** Adjusted mean C-reactive protein level (mg dl^−1^) by consumption category

*Consumption category*	*Onions*	*Tofu*	*Apples*	*Grapes*
Non-consumer	4.10	4.10	4.81	5.63
Infrequent (<1 × /month)	4.37	3.24	4.16	3.83
Monthly consumer (<3 × /month)	3.84	3.41	3.81	3.56
Weekly consumer (1–2 × /week)	3.66	2.65	3.28	4.09
Regular consumer (>3 × /week)	3.85	2.80	2.91	2.78
*p-trend*	0.002	0.004	0.001	0.02
				
			*Wine*	*Hot tea*
Non-consumer			4.30	5.71
Infrequent (1c/week or less)			3.44	4.43
Weekly, but not daily (2–6c/week)			3.39	3.84
Daily consumer (1c/day)			2.79	3.81
Multiple cups/day			4.09	3.26
*p-trend*			0.04	0.0002

CRP least-squared means presented are adjusted for sex, age, race, smoking status, SES, education, physical activity, BMI and total caloric intake.
